# Improved Mortality of Patients with Gastroschisis: A Historical Literature Review of Advances in Surgery and Critical Care from 1960–2020

**DOI:** 10.3390/children9101504

**Published:** 2022-09-30

**Authors:** Christina Georgeades, Alyssa Mowrer, Gezzer Ortega, Fizan Abdullah, Jose H. Salazar

**Affiliations:** 1 Division of Pediatric General Surgery, Children’s Wisconsin, Milwaukee, WI 53226, USA; 2 Department of Surgery, Medical College of Wisconsin, Milwaukee, WI 53226, USA; 3 Center for Surgery and Public Health, Department of Surgery, Brigham and Women’s Hospital, Harvard Medical School, Boston, MA 02115, USA; 4 Division of Pediatric General Surgery, Ann & Robert H. Lurie Children’s Hospital of Chicago, Chicago, IL 60611, USA; 5 Department of Surgery, Northwestern University Feinberg School of Medicine, Chicago, IL 60611, USA

**Keywords:** gastroschisis, pediatrics, mortality, mechanical ventilation, parenteral nutrition, pulmonary surfactant

## Abstract

The improved survival of gastroschisis patients is a notable pediatric success story. Over the past 60 years, gastroschisis evolved from uniformly fatal to a treatable condition with over 95% survival. We explored the historical effect of four specific clinical innovations—mechanical ventilation, preformed silos, parenteral nutrition, and pulmonary surfactant—that contributed to mortality decline among gastroschisis infants. A literature review was performed to extract mortality rates from six decades of contemporary literature from 1960 to 2020. A total of 2417 publications were screened, and 162 published studies (98,090 patients with gastroschisis) were included. Mortality decreased over time and has largely been <10% since 1993. Mechanical ventilation was introduced in 1965, preformed silo implementation in 1967, parenteral nutrition in 1968, and pulmonary surfactant therapy in 1980. Gastroschisis infants now carry a mortality rate of <5% as a result of these interventions. Other factors, such as timing of delivery, complex gastroschisis, and management in low- and middle-income countries were also explored in relation to gastroschisis mortality. Overall, improved gastroschisis outcomes serve as an illustration of the benefits of clinical advances and multidisciplinary care, leading to a drastic decline in infant mortality among these patients.

## 1. Introduction

Gastroschisis is a congenital paraumbilical abdominal wall defect that occurs in 3 to 4 cases per 10,000 live births and seems to be increasing in incidence [1,2,3,4,5]. The improved survival of patients with gastroschisis during the last century is one of the most notable medical advancements in the pediatric surgery population. Over the past 60 years, gastroschisis has evolved from a uniformly fatal disease process to a treatable condition with greater than 95% survival [6]. While most diseases have seen some improvement in prognosis over the past several decades, determining the specific interventions that have most significantly contributed to the mortality rate decline in gastroschisis infants remains largely unexplored. This historical literature review of gastroschisis analyzes the trend in mortality and the timing of advances in mechanical ventilation, use of the preformed silo, parenteral nutrition (PN), and pulmonary surfactant in regard to gastroschisis mortality. Other factors such as timing of delivery, presence of complex gastroschisis, and management strategies in low- and middle-income countries were also identified as factors contributing to the multifaceted management decisions for gastroschisis patients. These additional aspects of care exemplify not only the impact of clinical advancement on mortality rates, but also depict how certain populations of infants with gastroschisis and aspects of their care require unique considerations regarding survival.

## 2. Materials and Methods

The MEDLINE database was queried using PubMed with results limited to English language publications from 1960 to 2020. Using the search term “gastroschisis”, two authors screened all resulting articles. Manuscripts describing cohort, cross-sectional, or randomized studies were further scrutinized and those that reported mortality rates were included for full-text review. All articles before 1975 were scrutinized due to the paucity of literature during that period. Articles that described small cohorts (defined as <8 patients for publications prior to 1975, <30 patients from 1976–1999, and <75 patients from 2000 thereafter) were excluded. Additionally, excluded were studies focused solely on specific subpopulations of gastroschisis (e.g., patients with intestinal atresia, left sided-gastroschisis, maternal risk factors, and prenatal surveillance imaging) and studies reporting mortality in resource-limited settings. Since this was a review of the existing literature and did not include identifiable information, this study did not meet the definition of human subjects research and therefore no Institutional Review Board approval was necessary.

The gastroschisis mortality trend was plotted according to time, with the median of each study period (e.g., 1995 for a study reporting data collected from 1990 to 2000) on the x-axis and mortality rate on the y-axis. Publications that subdivided their mortality rates within discrete time periods were plotted as distinct data points. Using the United States Centers for Disease Control and Prevention report of infant mortality rate per 1000 live births, overall population-based infant mortality rates were plotted in parallel to allow comparison of the mortality trend over similar time periods [7]. The four interventions of mechanical ventilation, use of the preformed silo, PN, and pulmonary surfactant are also represented in graphical analysis. An arrow begins on the year of original publication in regard to its implementation and ending after 10 years, which was considered the approximate period of introduction.

## 3. Results

### 3.1. Improved Survival in Gastroschisis Infants

**Figure 1:**
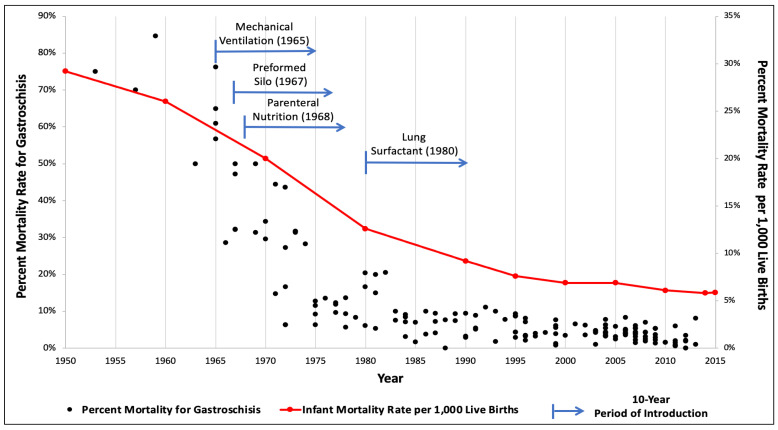
Gastroschisis and infant mortality rates compared over time with associated introduction of medical and surgical innovations.

A total of 2417 publications were screened, and 162 studies published between 1960 and 2020 were selected for inclusion in the analysis (Table S1). This process provided mortality rates for a combined population of 98,090 infants with gastroschisis. Mortality rates ranged from 0 to 85% (Figure 1). Reported mortality has consistently been less than 20% since 1983 and less than 10% after 1993. Gastroschisis infants now largely carry a mortality rate of <5%. For the landmark advances, mechanical ventilation was introduced in 1965, use of a preformed silo in 1967, PN in 1968, and surfactant therapy in 1980 [8,9,10,11].

Over the last 60 years, advances in modern medicine have led to substantial improvements in the overall survival of neonates. As these developments occurred, the clinical management of gastroschisis progressed such that mortality is far less common. The present study extracted mortality rates from six decades of contemporary literature from 1960 to 2020. This is the most extensive report focusing on death rates from gastroschisis due to the length of time analyzed and the number of studies evaluated. When the data was plotted graphically, there was a pronounced trend towards decreased gastroschisis mortality as a function of time. Furthermore, when simultaneously displayed with population-based infant mortality rates, there was a striking similarity in the shape of the curves. Lastly, this review illustrated the inflection points of the gastroschisis and overall infant mortality curves as influenced by the introduction of four scientific advances: mechanical ventilation, use of a preformed silo, PN, and pulmonary surfactant.

### 3.2. Implementation of Clinical Interventions

The first era is closely related to the introduction of mechanical ventilation suitable for neonates. Many patients with gastroschisis require ventilatory support during their repair or hospital stay. In newborns, the first case of prolonged ventilation was reported in 1953, but it required large face masks or tracheostomies [12]. It was not until 1965 that adequate endotracheal tubes were available and Thomas et al. reported management of a series of 18 newborns with severe respiratory distress, of which 11 survived [8]. This advance likely had immediate effects on cause-specific and overall infant mortality rates. In particular, when premature gastroschisis infants experience respiratory distress after being born, either electively or spontaneously, they now have a means of survival that they did not have before. For neonates with gastroschisis, the supportive care provided by mechanical ventilation has become a cornerstone of care that ultimately assists with clinical progression for infants born with gastroschisis [13].

It is unclear when the first successful surgical repair of gastroschisis occurred, since the condition was not clearly differentiated until 1953 [14]. Prior to the 1950s, pediatric surgeons had little guidance apart from scattered reports of individual cases. Once the surgical treatment with primary repair was well established, the primary pathophysiologic mechanism affecting postoperative mortality was intestinal failure and increased intra-abdominal pressure leading to pulmonary insufficiency, acute kidney injury, and cardiac failure from diminished venous return. In 1967, Schuster described the novel use of a synthetic material to construct a temporary extracorporeal reservoir, allowing for a progressive reduction in viscera into the abdominal cavity [9]. This technique was later adapted and improved by Shermeta and Haller into a preformed transparent silo [15]. Therefore, this prevented the complications that occur from increased intra-abdominal pressure.

Preformed silos are typically used for cases not amenable to primary closure and instead need staged, or delayed, closure. Currently, controversy still exists when choosing the method for gastroschisis repair and practice varies by institution. In general, primary closure is attempted when the hemodynamic status of the patient and size of the defect permits successful performance. A staged repair is more commonly used for larger defects, significant bowel matting, or to allow for preoperative resuscitation. The creation and implementation of preformed silos has not only led to a reduction in ventilator days, rate of infection, and time to first feed, but also has resulted in improved survival for patients that may not tolerate primary closure [16,17].

The next intervention leading to improved outcomes in gastroschisis was the introduction of PN. The use of PN has been one of the most important advances in neonatal care for infants with gastroschisis [18]. Nutritional status often complicates the care of these patients as the exposure of bowel to amniotic fluid is associated with prolonged dysmotility and ileus [19,20,21]. The first infant to benefit from PN was a newborn girl with near-total small bowel atresia treated at Children’s Hospital of Philadelphia in 1968 [10]. Unable to be fed by mouth, she faced a poor prognosis but survived for 22 months receiving nutrition primarily intravenously [10]. The use of PN was refined and rapidly disseminated with multiple reports documenting a survival advantage throughout the 1970s [22]. Gastroschisis patients are one of many populations that benefited from this advance. Until the introduction of PN, many neonates with gastroschisis died from malnutrition and subsequent infectious complications due to the prolonged ileus that can occur [18]. Additionally, neonates with disease processes such as necrotizing enterocolitis, bowel perforation, and bowel atresia—all associated with gastroschisis—that preclude enteral feeding are now routinely supported by this life-saving therapy.

The final phase of improved outcomes in gastroschisis infants can be attributed to the introduction of pulmonary surfactant into clinical practice. The advantages of surfactant are observed primarily in preterm neonates, which is highlighted in this patient population given that 46% to 61% are born preterm [23]. In 1980, Fujiwara et al. conducted the first trial of surfactant therapy in 10 Japanese infants and showed an astounding improvement in oxygenation [11]. Multiple trials followed, showing a significant benefit that drove widespread adoption of this therapy. The introduction of pulmonary surfactant to preterm neonates with respiratory distress syndrome has led to not only improved outcomes, but also decreased mortality [24]. As a result, those born with gastroschisis prematurely have consequently benefited, leading to improved survival specifically in this population.

### 3.3. Current and Future Considerations

#### 3.3.1. Timing of Delivery

Though mortality rates in gastroschisis infants have greatly declined over the past decades, there are still unknown aspects of management that may potentially impact survival. Though controversial, determining the timing of delivery in pregnancies with fetal gastroschisis is important for minimizing the risk of prenatal and postnatal mortality. Some studies support delivery at term gestational age due to better neonatal outcomes while others advocated for elective delivery ≤37 weeks gestation due to the increased risk of stillbirth and morbidity with expectant management [25,26,27]. Delivery at term potentially avoids complications associated with pre-term birth, such as respiratory distress requiring prolonged ventilation and oxygen therapy, brain damage, and retinopathy of prematurity [6,28]. However, the rate of fetal demise in pregnancies related to gastroschisis is estimated to be as high as 12.5% and is thought to potentially be due to cytokine-mediated inflammatory responses, cardiovascular compromise, volvulus, or umbilical cord compression from bowel dilation [6,25,29]. Existing data are largely comprised of cohort studies with small sample sizes at single-institutions and further research is needed [28].

Determination of the optimal timing of delivery is critical for continuing the trend of improved survival for gastroschisis infants. It is important to note that the invention and implementation of the aforementioned advances, such as mechanical ventilation and pulmonary surfactant, have led to the ability to consider elective preterm delivery due to their life-sustaining impact for premature neonates. This consideration for improving outcomes would not have been possible a few decades ago.

#### 3.3.2. Complex Gastroschisis

Complex gastroschisis is defined as the presence of bowel atresia, perforation, necrosis, and/or volvulus while the absence of these pathologies defines simple gastroschisis [30,31,32,33]. Complex gastroschisis affects 17% of infants born with the condition, and mortality in this specific population is 7.6 times higher than those born with simple gastroschisis [32]. These neonates not only have higher mortality, but they also experience more complications and increased length-of-stay [33]. In particular, short bowel syndrome is a complication that is specific to this population, which portends a mortality rate as high as 37.5% due to causes such as hepatic failure, sepsis, or multi-organ failure [34]. Necrotizing enterocolitis is also much higher in this population.

Another important consideration is that though interventions such as PN and mechanical ventilation have had immense positive impact on infants with severe pathology, there are risks and complications that are associated with long-term use, such as infection, necrotizing enterocolitis, neurologic impairment, pulmonary complications, and liver dysfunction [35,36]. Though these complications can affect infants with simple gastroschisis, those with complex gastroschisis may require longer use of such interventions. Therefore, this subset of patients is placed at a paradoxical higher risk of complications from the same interventions that have dramatically improved outcomes and mortality in the overall gastroschisis population.

To optimize the outcomes and minimize complications of patients with complex gastroschisis, targeted approaches must be utilized and new interventions considered. A multi-disciplinary approach to prenatal counseling, risk stratification, and management of complex cases is vital in this consideration [32,33]. Though potential predictive factors of complex gastroschisis have been identified with the use of prenatal imaging, refinement of prenatal evaluation of complex gastroschisis is still needed [32,37]. Furthermore, fetal surgery has also been more recently proposed, though further data and investigation is needed regarding this innovative intervention [38,39].

#### 3.3.3. Low- and Middle-Income Countries

The significant decline in mortality rates for infants born with gastroschisis due to the aforementioned clinical advances and resources has primarily occurred in high-income countries. Survival for neonates in low- and middle-incomes countries has not demonstrated a similar decline due to a multitude of factors. Mortality rates in these countries are as high as 50–100%, with one study showing the greatest disparity in survival between low-income (10% survival), middle-income (68.1% survival), and high-income (98.6% survival) countries for infants with gastroschisis compared to infants with other congenital conditions [40,41]. Factors shown to be associated with decreased survival include lack access to mechanical ventilation or PN [41]. Additionally, utilization of preformed silos and pulmonary surfactant is minimal in lower income countries due to expense and/or lack of knowledge on how to properly execute the intervention [42,43]. Other barriers, such as lack of prenatal diagnosis, limited facilities and resources, poor availability of operating rooms and safe anesthesia, and delays in healthcare access, also negatively influence the disparities in mortality for gastroschisis neonates [40]. Recognition of these inequalities and provision of access to important clinical interventions is critical for decreasing mortality rates of infants born with gastroschisis in low- and middle-income countries.

## 4. Conclusions

This literature review provides a broad overview of factors influencing the marked decrease in mortality for patients with gastroschisis. The introduction of mechanical ventilation, preformed silos, PN, and pulmonary surfactant have played crucial roles in this trend, and infants born with gastroschisis now carry an overall mortality risk of less than 5%. Determination of the optimal timing of delivery may be a next step in continuing to decrease mortality and improve outcomes. However, certain populations, such as infants born with complex gastroschisis and/or in low- and middle-income countries continue to have disproportionately lower survival. Recognition of these disparities and the potential interventions needed are necessary steps to ultimately address these inequalities. Overall, the improved survival during the last century for gastroschisis infants due to advancements in critical and surgical care is one of the most notable success stories in the history of pediatrics and pediatric surgery. Ongoing research and advancements in clinical interventions are important for the continued decline in mortality and improved outcomes for infants born with gastroschisis.

## Data Availability

Not applicable.

## Supplementary Materials

The following supporting information can be downloaded at: https://www.mdpi.com/article/10.3390/children9101504/s1, Table S1: List of publications and corresponding mortality rates for gastroschisis from articles published from 1960–2020.
