# Supplementary material for: Improved Mortality of Patients with Gastroschisis: A Historical Literature Review of Advances in Surgery and Critical Care from 1960–2020
Source: Children (Basel). 2022 Sep 30;9(10):1504. doi: 10.3390/children9101504 (PMC9600704; doi:10.3390/children9101504)
Supplement: Supplementary file 1 [file children-09-01504-s001.zip › Table S1.pdf]

Table S1. List of publications and corresponding mortality rates for gastroschisis from articles published from 1960 - 2020

First Author	Country of Study	Year of Publication	Time of Data Collection	Time Charted	Total Cases	Deaths	Mortality Rate
King	USA	1980	1947-1959	1953	8	6	75.0%
Hollabaugh	USA	1973	1947-1967	1957	20	14	70.0%
Rickham	UK	1963	1955-1963	1959	13	11	84.6%
Savage	Australia	1971	1956-1969	1963	8	4	50.0%
Mabogunje	USA	1984	1960-1970	1965	37	24	64.9%
King	USA	1980	1960-1969	1965	30	17	56.7%
Aaronson	UK	1977	1959-1970	1965	21	16	76.2%
Mahour	USA	1973	1960-1970	1965	23	14	60.9%
Lindham	Sweden	1987	1956-1975	1966	14	4	28.6%
Klein	USA	1981	1954-1979	1967	59	19	32.2%
Lewis	USA	1973	1962-1972	1967	31	10	32.3%
Gilbert	USA	1972	1963-1971	1967	17	8	47.1%
Rangarathnam	USA	1969	1966-1968	1967	8	4	50.0%
Knutrud	Norway	1979	1960-1978	1969	32	10	31.3%
Bryant	USA	1970	1968-1969	1969	8	4	50.0%
Hasse	Germany/ Switzerland/Austria	1979	1962-1977	1970	259	89	34.4%
Hollabaugh	USA	1973	1967-1972	1970	27	8	29.6%
Schwaitzberg	USA	1982	1961-1981	1971	34	5	14.7%
Wesselhoeft	USA	1972	1969-1972	1971	18	8	44.4%
Fonkalsrud	USA	1993	1965-1979	1972	32	2	6.3%
Kekomäki	Finland	1982	1964-1980	1972	39	17	43.6%
Fonkalsrud	USA	1980	1965-1979	1972	32	2	6.3%
Stringel	Canada	1979	1967-1977	1972	44	12	27.3%
Raffensperger	USA	1974	1970-1973	1972	24	4	16.7%
Mabogunje	USA	1984	1970-1975	1973	32	10	31.3%
Ein	Canada	1980	1969-1976	1973	44	14	31.8%
Aaronson	UK	1977	1971-1975	1973	22	7	31.8%
Rubin	Canada	1978	1970-1977	1974	46	13	28.3%
Swartz	USA	1986	1967-1983	1975	104	12	11.5%
Canty	USA	1983	1967-1983	1975	54	5	9.3%
Mayer	USA	1980	1971-1979	1975	47	6	12.8%
King	USA	1980	1970-1979	1975	64	4	6.3%
Kirk	USA	1983	1970-1981	1976	74	10	13.5%
Blakelock	New Zealand	1997	1969-1995	1977	42	5	11.9%
Luck	USA	1985	1970-1984	1977	106	13	12.3%
Schwartz	USA	1983	1970-1983	1977	31	3	9.7%
Davies	UK	1997	1972-1984	1978	35	2	5.7%

Di Lorenzo	Canada	1987	1971-1985	1978	59	8	13.6%
Mabogunje	USA	1984	1975-1980	1978	32	3	9.4%
Novotny	USA	1993	1972-1985	1979	36	3	8.3%
Tunell	USA	1995	1975-1984	1980	66	4	6.1%
Oldham	USA	1988	1974-1986	1980	54	11	20.4%
Othersen	USA	1986	1975-1985	1980	36	6	16.7%
Shah	USA	1991	1972-1989	1981	74	11	14.9%
Lindham	Sweden	1987	1976-1985	1981	19	1	5.3%
Blane	USA	1985	1978-1983	1981	30	6	20.0%
Nagaya	Japan	1993	1970-1991	1982	39	8	20.5%
Stringer	UK	1991	1977-1989	1983	40	4	10.0%
Caniano	USA	1990	1979-1986	1983	80	6	7.5%
Snyder	USA	2001	1969-1999	1984	199	17	8.5%
Snyder	USA	1999	1969-1999	1984	185	17	9.2%
Moretti	USA	1990	1978-1989	1984	56	4	7.1%
Sipes	USA	1990	1979-1989	1984	31	1	3.2%
Nicholls	USA	1993	1980-1989	1985	43	3	7.0%
Sawin	USA	1992	1980-1989	1985	116	2	1.7%
Fonkalsrud	USA	1993	1980-1992	1986	52	2	3.8%
Bryant	USA	1989	1984-1988	1986	30	3	10.0%
Snyder	USA	2005	1970-2003	1987	222	16	7.2%
Cusick	UK	1997	1982-1992	1987	63	6	9.5%
Tawil	Australia	1995	1980-1993	1987	49	2	4.1%
Ramsden	UK	1997	1982-1993	1988	66	5	7.6%
Novotny	USA	1993	1986-1990	1988	33	0	0.0%
Burge	UK	1997	1982-1995	1989	54	4	7.4%
Coughlin	USA	1993	1986-1991	1989	32	3	9.4%
Dunn	USA	1999	1985-1995	1990	60	2	3.3%
Boyd	UK	1998	1985-1995	1990	34	1	2.9%
Adra	USA	1996	1986-1994	1990	42	4	9.5%
Salihu	USA	2004	1983-1999	1991	354	18	5.1%
Hwang	USA	2004	1982-1999	1991	79	7	8.9%
Quirk	USA	1996	1987-1995	1991	56	3	5.4%
Friedmacher	Austria	2014	1975-2008	1992	108	12	11.1%
Jayanthi	UK	1998	1990-1996	1993	60	6	10.0%
Simmons	USA	1996	1990-1995	1993	55	1	1.8%
Driver	UK	2000	1991-1997	1994	91	7	7.7%
Cherian	UK	2006	1990-1999	1995	116	5	4.3%
Puligandla	Canada	2004	1990-2000	1995	113	10	8.8%
Singh	Australia	2003	1990-2000	1995	181	8	4.4%
Molik	USA	2001	1992-1997	1995	103	9	8.7%

Rinehart	USA	1999	1992-1998	1995	32	3	9.4%
Fisher	UK	1996	1994-1995	1995	34	1	2.9%
Baud	Canada	2013	1980-2011	1996	153	5	3.3%
Kronfli	UK	2010	1983-2009	1996	179	4	2.2%
Hadidi	Germany	2008	1986-2006	1996	86	3	3.5%
Arnold	USA	2007	1988-2003	1996	4344	152	3.5%
Salihu	USA	2003	1992-1999	1996	308	25	8.1%
Nembhard	USA	2001	1995-1997	1996	127	9	7.1%
Carnaghan	UK	2014	1992-2012	1997	246	8	3.3%
Ergün	USA	2005	1992-2002	1997	75	3	4.0%
Kidd	USA	2003	1993-2002	1998	118	5	4.2%
Harris	Australia	2014	1992-2005	1999	103	4	3.9%
Durfee	USA	2013	1990-2008	1999	98	6	6.1%
Kassa	Sweden	2011	1989-2009	1999	79	6	7.6%
Banyard	USA	2010	1990-2008	1999	235	2	0.9%
Payne	USA	2009	1990-2007	1999	155	2	1.3%
Charlesworth	UK	2007	1993-2005	1999	110	6	5.5%
Alvarez	USA	2007	1996-2003	2000	9459	333	3.5%
Kong	Australia	2016	1992-2009	2001	336	22	6.5%
Clark	USA	2009	1997-2007	2002	2507	89	3.6%
Fillingham	UK	2008	1997-2006	2002	130	8	6.2%
Carnaghan	Canada/UK	2020	1992-2014	2003	500	23	4.6%
Sacks	USA	2016	1999-2007	2003	1537	74	4.8%
Clark	USA	2011	1997-2008	2003	2749	115	4.2%
Jensen	USA	2009	1998-2007	2003	91	1	1.1%
Raitio	Finland	2020	1993-2014	2004	235	18	7.7%
Anderson	USA	2018	1995-2012	2004	2527	104	4.1%
Feldkamp	USA	2016	1997-2011	2004	316	14	4.4%
Cain	USA	2014	1998-2009	2004	324	11	3.4%
Minutillo	Australia	2013	1997-2010	2004	117	5	4.3%
Abdelhafeez	Ireland	2011	1999-2009	2004	128	8	6.3%
Tam Tam	USA	2011	2000-2008	2004	104	4	3.8%
Snyder	USA	2011	1999-2009	2004	167	9	5.4%
Alali	USA	2011	1998-2009	2004	87	3	3.4%
Lansdale	UK	2009	2001-2007	2004	150	5	3.3%
Corey	USA	2014	1997-2012	2005	4687	143	3.1%
Weil	USA	2012	2000-2009	2005	203	12	5.9%
Orion	USA	2011	2000-2009	2005	80	2	2.5%
Sparks	USA	2017	2005-2006	2006	819	68	8.3%
Abdelhafeez	USA	2015	2000-2012	2006	142	7	4.9%
Bucher	USA	2012	2001-2010	2006	118	6	5.1%

Alfaraj	Canada	2011	2001-2010	2006	98	4	4.1%
Lao	USA	2010	2003-2008	2006	2490	90	3.6%
Boutros	Canada	2009	2005-2007	2006	192	9	4.7%
Weinsheimer	Canada	2008	2005-2006	2006	99	4	4.0%
Skarsgard	Canada	2008	2005-2006	2006	100	4	4.0%
Lopez	USA	2019	1999-2014	2007	2925	158	5.4%
Koehler	USA	2018	2000-2014	2007	135	6	4.4%
Koehler	USA	2017	2000-2014	2007	171	9	5.3%
Carnaghan	Canada	2016	2000-2014	2007	217	5	2.3%
Nasr	Canada	2012	2005-2008	2007	395	15	3.8%
Cowan	Canada	2012	2005-2008	2007	409	13	3.2%
Jansen	Canada	2012	2005-2009	2007	407	25	6.1%
Bradnock	UK/Ireland	2011	2006-2008	2007	302	12	4.0%
Owen	UK	2010	2006-2008	2007	393	6	1.5%
Mills	Canada	2010	2005-2008	2007	249	11	4.4%
Dubrovsky	USA	2017	2003-2012	2008	7769	336	4.3%
Youssef	United States/Canada	2016	2003-2012	2008	5216	220	4.2%
Emami	Canada	2015	2005-2011	2008	565	11	1.9%
Charlesworth	UK	2014	1993-2012	2008	156	5	3.2%
Aljahdali	Canada	2013	2005-2011	2008	570	40	7.0%
Safavi	Canada	2012	2005-2010	2008	402	10	2.5%
Hawkins	USA	2020	2005-2013	2009	566	30	5.3%
Palatnik	USA	2020	2000-2017	2009	206	11	5.3%
Raymond	USA	2020	2005-2013	2009	566	30	5.3%
Gupta	USA	2018	2006-2012	2009	3741	85	2.3%
Youssef	Canada	2017	2005-2013	2009	700	10	1.4%
Song	USA	2017	2006-2012	2009	3846	142	3.7%
Youssef	United States/Canada	2016	2005-2013	2009	695	20	2.9%
Lusk	USA	2014	2007-2012	2010	191	3	1.6%
Overcash	USA	2014	2007-2012	2010	191	3	1.6%
Brebner	Canada	2020	2009-2013	2011	4803	287	6.0%
Arnold	USA	2018	2009-2012	2011	146	3	2.1%
Girsen	USA	2016	2008-2013	2011	108	1	0.9%
Girsen	USA	2015	2008-2013	2011	112	1	0.9%
Murthy	USA	2014	2010-2012	2011	602	3	0.5%
Savoie	USA	2014	2008-2013	2011	524	8	1.5%
Witt	USA	2019	2007-2017	2012	97	0	0.0%
Hong	USA	2019	2009-2015	2012	4663	90	1.9%
Bhatt	USA	2018	2010-2014	2012	8872	311	3.5%

Fraga	USA	2018	2008-2016	2012	125	0	0.0%
Fullerton	USA	2017	2009-2014	2012	4420	97	2.2%
Fujiogi	Japan	2018	2010-2016	2013	247	20	8.1%
Gurien	USA	2017	2012-2014	2013	627	7	1.1%
